# Roles of Probiotics and Prebiotics in Colon Cancer Prevention: Postulated Mechanisms and In-vivo Evidence

**DOI:** 10.3390/ijms9050854

**Published:** 2008-05-20

**Authors:** Min-Tze Liong

**Affiliations:** Food Technology Division, School of Industrial Technology, Universiti Sains Malaysia, 11800 Minden, Penang, Malaysia; E-mail: mintze.liong@usm.my; Tel-: +604-653-2114; Fax: +604-657-3678

**Keywords:** probiotic, prebiotic, synbiotic, colon cancer, mechanism

## Abstract

Probiotics are live bacteria that could exert health beneficial effects upon consumption. In additional to their conventional use as gut modulators, probiotics are investigated for their role to prevent cancer. In-vivo and molecular studies have demonstrated encouraging outcomes, mainly attributed to its antimicrobial effects against carcinogen-producing microorganisms, antimutagenic properties, and alteration of the tumor differentiation processes. Prebiotics are indigestible food components that could promote the growth of beneficial bacteria including probiotics. Present studies have suggested that prebiotics also possess protective effect against colon carcinogenesis, mainly attributed to the production of short chain fatty acids upon its fermentation by gut microflora, and alteration of gene-expressions in tumor cells. Synbiotic (combination of probiotic and prebiotic) has been found to exert a synergistic effect in improving colon carcinogenesis compared to when both were used individually. This paper highlights the colon cancer preventive effects by probiotics, prebiotics and synbiotics. In addition, the controversial outcomes on the insignificant effect of these food adjuncts will be discussed.

## 1. Introduction

Probiotics are defined as ‘live microorganisms which when administered in adequate amounts confer a health benefit on the host’ [1]. Certain strain of bacteria haven been discovered over the years to have probiotic properties, mainly consisting of lactic acid producing bacteria (lactobacilli, streptococci, enterococci, lactococci, bifidobacteria), *Bacillus* and fungi such as *Saccharomyces* and *Aspergillus*. Probiotics is well-known for its roles in modulating a healthier gut. Now, probiotics are found to exert other health advantages such as improving lactose intolerance, increasing humoral immune responses, biotransformation of isoflavone phytoestrogen to improve post-menopausal symptoms, bioconversion of bioactive peptides for antihypertension, and reducing serum cholesterol level [2]. A prebiotic is a non-digestible food ingredient that beneficially affects the host by selectively stimulating growth, activity, or both of one or a limited number of bacterial species already resident in the colon [3]. To exhibit such effects, a prebiotic must neither be hydrolysed nor absorbed in the upper part of the gastrointestinal tract, and must be selective for one or a limited number of potentially beneficial bacteria such as probiotics that are residing in the colon [4]. Another possibility of gut microflora management is the use of synbiotics, where probiotics and prebiotics are used in combination.

Colorectal cancer represents a major public health problem accounting for over 1 million cases and about half a million deaths worldwide [5]. Survival from colon cancer at 5 years has been found to vary demographically and estimated to be 65% in North America, 54% in Western Europe, 34% in Eastern Europe, and 30% in India [6]. Although chemotherapy and radiotherapy have been applied as the surgical adjuvant treatments of colon cancer, they vary in success rates for local recurrence, disease-free survival, and overall survival [7]. In addition, these treatments may present some side effects such as an increased risk for infections, hair loss, fatigue, lip sores, nausea, vomiting, diarrhea, and bloody stools. Diet interventions and natural bioactive supplements have now been extensively studied to reduce the risks of colon cancer, as a cause of prevention instead of cure. Postulated mechanisms include reduction in the activity of several cancer causing bacteria, desmutagenic and anticarcinogenic properties [4,5]. Prebiotics, being indigestible, have been associated with improved bowel functions and metabolisms of the distal colon, including a reduced risk of colon cancer. Lower tumor numbers have been observed in rats treated with carcinogens when they were fed cereal bran [8]. One of the main postulated mechanisms is the production of short chain fatty acids especially butyrate via the fermentation of prebiotics by gut flora. In addition, synbiotic products have also been found to exert increased benefits compared to the administration of either probiotic or prebiotic alone.

This present review highlights some of the roles of probiotics, prebiotics and synbiotics on the prevention of colon cancer. Hypotheses and in-vivo evidence will be provided and some controversial results will be discussed.

## 2. Protective Roles of Probiotics on Colon Cancer

Early studies had indicated that the general population of intestinal bacteria is associated with initiation of colon cancer via the production of carcinogens, co-carcinogens, or pro-carcinogens [9]. A previous study has reported that only 20% of germ-free animals were shown to develop chemically induced colon tumors, as compared with 93% of their counterparts with a normal flora [10]. Using azoxymethane-induced aberrant crypt foci in rats, Reddy *et al.* [11] found that a stimulated growth of bifidobacteria in the colon could lead to the inhibition of colon carcinogenesis. The authors suggested that the inhibition of aberrant crypt foci and crypt multiplicity was attributed to the pH-lowering effect of bifidobacteria in the colon, which subsequently inhibited the growth of *E. coli* and clostridia. A decrease in growth of such pathogenic microorganisms may also produce the modulation of bacterial enzymes such as beta-glucuronidase that can convert pro-carcinogens to proximate carcinogens [12].

Such anticarcinogenic properties have also been studied at a molecular level. There are fifty-seven cytochrome P450 encoded in the human genome, mainly catalyzing the metabolism of steroids, bile acids, eicosanoids, drugs and xenobiotic chemicals [13]. However, some of the P450s are also active carcinogens. Past epidemiological researches have shown increased risk of colon cancer in individuals with high P4501A2 activity. The metabolic activation of food-borne heterocyclic amines to colon carcinogens in humans is hypothesized to occur via N-oxidation followed by O-acetylation to form the N-acetoxy arylamine that binds to DNA to give carcinogen-DNA adducts. These steps are catalyzed by hepatic cytochrome P4501A2 and acetyltransferase-2 (NAT-2), respectively [14]. It has been postulated that probiotics such as *Bifidobacterium* could lower the risks of colon cancer, by producing metabolites that could affect the mixed-function of P450s and subsequently affect the conversion of azoxymethane from proximate to ultimate carcinogen [15]. These had led to the suggestion that probiotic could suppress colon cancer.

In-depth investigations have also showed that cultured milk possess desmutagenicity and this activity increase with increasing numbers of viable cells, indicating that probiotic play an important role in the inhibition of mutagenicity [16]. Thyagaraja and Hosono [17] found that probiotic isolated from “idly”, a traditional cereal pulse product of India could exert desmutagenicity on various spice mutagens, heterocyclic amines and aflatoxins. Subsequent studies on the desmutagenicity properties of probiotic suggested that the desmutagenic substances may reside in the cellular envelope of the bacterial cell wall [10]. Cell wall preparation of *B. infantis* was found to inhibit tumor activity in mouse peritoneal cells in-vitro [18], while cell wall preparation of heat-killed *L. casei* (LC9018) was found to induce immunity against tumor induction in a randomized, controlled and comparative study involving 223 patients with stage III cervical cancer. The antitumor effects were found to be due to the activation of macrophage by LC9018 [19]. Also, mutagens were suggested to be bound to the cell wall of probiotics. This has been supported by previous studies that have found binding properties by fractions of the cell wall skeleton of probiotics on mutagens [20] and the binding of heterocyclic amines by intestinal probiotics [21]. In addition, whole cells of bifidobacteria have also been found to bind with the ultimate carcinogen methylazoxymethanol [12] and mutagen-carcinogen 3-amino-1,4-dimethyl-5*H*-pyrido[4,3-*b*]indole [22], thus physically removing it via feces and subsequently minimizing its absorption into the intestinal lumen.

Other studies have postulated that probiotics possess colon cancer protective effects by altering the differentiation process of tumor cells. Using a cultured human colon cancer cell line (HT-29), Baricault *et al.* [23] studied the effect of fermented milks on colon cancer cell growth. Milks were fermented with individual strains of *Lactobacillus helveticus, Bifidobacterium, L. acidophilus* or a mix of *Streptococcus thermophilus* and *L. delbrueckii* subsp. *bulgaricus*. The HT-29 cells were subsequently added into the fermented milk, and the authors found that 10–50% of the HT-29 cells showed a decrease in growth. Further analyses revealed that the specific activities of specific marker for HT-29 cell differentiation such as the dipeptidyl peptides were increased. The authors suggested that the tumor cells entered a differentiation process leading to lower growths.

Using male weanling F344 rats, Singh *et al.* [10] evaluated the effect of *B. longum* on colon carcinogenesis. Results from their 40-week study demonstrated that dietary administration of lyophilized cultures of *B. longum* resulted in a suppression of colon tumor incidence and tumor multiplicity and also reduced tumor volume. Analyses on intermediate biomarkers revealed that ingestion of *B. longum* inhibited azoxymethane-induced cell proliferation via a reduction in ornithine decarboxylase (ODC) activity. ODC is involved in the biosynthesis of polyamines that causes cell proliferation, differentiation and macromolecular synthesis. Increased ODC activity has been associated with increasing colon adenomas and carcinomas which indicate a hyperproliferative state of the colonic mucosa [24]. In addition, Singh *et al.* [10] also found that the strong antitumor activities and the reduction in the outcome of tumor were attributed to a reduced expression of *ras*-p21 oncoprotein when rats were fed *B. longum*. Activation of *ras* proto-oncogens could induce a malignant phenotype in colonic cells [25]. The malignant potential of *ras* genes has been associated with a mutational activation in codons 12, 13 or 16 [26]. Such a mutation is most observed in the human colon tumors, in aberrant crypt foci, colon adenomas and carcinomas [10].

## 3. Roles of Prebiotics and Synbiotics

Most of the protective effects of prebiotics on colon cancer have emphasized on the oligofructose-based prebiotics such as fructooligosaccharides and inulin. In one of the animal trial conducted (trial of 31 weeks), Femia *et al.* [27] reported that the protective effect of probiotics on azoxymethane-induced carcinogenesis was less compared to the effects of prebiotics (oligofructose-inulin). Although the authors found that probiotics could reduce malignant tumors in the colon of rats, their effect was insignificant statistically, while colonic proliferation was lower when rats were fed the prebiotics. The authors subsequently conducted further studies on the expression of genes codifying enzymes involved in colon carcinogenesis processes. Glutathione *S*-transferase and GST placental enzyme pi type were found to be expressed lower when rats were fed the prebiotic individually and when fed in combination with *Lactobacillus rhamnosus* GG and *Bifidobacterium lactis* Bb12. In addition, the inducible nitric oxide synthase, found to play an important role in colon tumor growth and progression [28], was also depressed in the tumors from rats in the prebiotic group. The authors also evaluated the levels of cyclooxygenase-2, an enzyme found to be up-regulated in cancers [29], and cyclooxygenase-2 inhibitors which are often associated with chemopreventive activities. Cycloxygenase-2 expression was found to be increased in the tumours of the control rats but not in those fed prebiotics. Although the exact mechanisms remain unknown, Femia *et al.* [27] postulated that prebiotic reduced carcinogenesis via modification of gene-expressions.

The fermentation of prebiotics in the colon often generates short chain fatty acids (SCFA). Considering that butyrate is not a common end-product from the fermentation of lactobacilli and bifidobacteria, its production would originate from the fermentation by other intestinal flora. It has been found that butyrate is produced in the colon at varying concentrations depending on the type of prebiotics [30]. Although the production of butyrate is approximately 5% of total SCFA, it is of particular interest because butyrate has been found to induce differentiation of phenotype including colorectal tumor cells [31]. The reduction of colonic cell proliferation and induction of differentiation in colonic epithelial cells have now led to increased clinical trials of butyrate in the treatment of ulcerative colitis [11]. In addition, sodium butyrate was revealed as a powerful inhibitor of growth and inducer of phenotype differentiation and apoptosis and is considered to exert beneficial effects in reducing risk factors involved in the etiology of colon cancer and adenoma development [32]. Treptow-van Lishault *et al.* [33] found that the fermentation of gut bacteria on a retrograded, high amylose starch had produced butyrate that may increase the detoxification of both electrophilic products and compounds associated with oxidative stress. The enzyme induction by butyrate, or by the microflora and increased activity by prebiotics, may be an important mechanism of protection against carcinogen-enhanced colon cancer [34].

The combination of suitable prebiotics with probiotic/s has been found to enhance the survival and activity of the organism, both in in-vitro and in-vivo experiments, for example a fructooligosaccharide in conjunction with a *Bifidobacterium* strain or lactitol in conjunction with *Lactobacillus* [3]. The combination of prebiotic and probiotic has synergistic effects because in addition to promoting growth of existing strains of beneficial bacteria in the colon, synbiotics also act to improve the survival, implantation and growth of newly added probiotic strains. The combination of *Bifidobacterium* and oligofructose synergistically retarded colon carcinogenesis in rats compared to when both were given individually [35]. In a randomized, double-blind and placebo-controlled trial, Rafter *et al.* [36] evaluated the effect of synbiotic on reducing cancer risk factors in 37 colon cancer patients and 43 polypectomized patients. The synbiotic contained *Lactobacillus rhamnosus* GG and *Bifidobacterium lactis* Bb12 as the probiotics and oligofructose-enriched inulin as the prebiotic. The authors found that certain colorectal cancer intermediate biomarkers can be altered via synbiotic intervention, where colorectal proliferation and the capacity of fecal water to induce necrosis in colonic cells were reduced. In addition, polypectomized patients showed improved epithelial barrier functions. The authors conducted genotoxicity assays using the colonic biopsy samples, and found that the exposure to genotoxins in polypectomized patients decreased at the end of the intervention period. Although the exact mechanisms of these remain unknown, the authors postulated that the synbiotic intervention had contributed to the alterations in the composition of the colonic bacterial ecosystem, and subsequently the metabolic activity of the colon.

## 4. Controversial Findings

Despite all the claimed colon cancer protective effects, not all investigations support this outcome, where *in-vivo* trials have given inconsistent data. Such contradictory results obtained may be related to the complexity of carcinogenesis, experimental design, complications in obtaining the appropriate sample sizes, variation in the type of probiotic strains and variations in the tumor stages of subjects. In addition, the desired protective effects have also been found to depend on the dose of probiotics administered. A protective effect of *L. casei* against carcinogen-induced lesions in colon cells of rats were observed, when the probiotics were administered at a level of 1 × 10^10^ bacteria in 10 mL NaCl/kg body weight. Similarly, a single dose of living *L. acidophilus*, *L. gasseri*, *L. confusus*, *Streptococcus thermophilus*, *B. breve*, and *B. longum* was found to prevent MNNG-induced DNA damage in the colon. However, a reduced bacterial dose of 50% or 90% had resulted in the loss of the carcinogen protection effects [37]. This may be attributed to the probiotic dose-dependent stimulation of gut immune cells to release inflammatory and regulatory cytokines such as gamma interferon, interleukin-12 (IL-12), IL-14 and IL -10 [38].

Moore and Moore [39] evaluated the relationships between intestinal flora of different nationalities and colon cancer. Subjects from Japan (n=22) and South Africa (n=16) were grouped as low-risk populations due to their minimal daily intake of red meat, while the US Caucasians (n=17) were grouped as a high-risk population. The authors reported that *B. longum* and *B. angulatum* were positively associated with a high risk of colon cancer, where their populations were found to increase with increased risk of colon cancer. In addition, increased numbers of *Bifidobacterium* was detected in the flora of the high-risk populations, a contrary to some suggestions by commercial companies that ingestion of *Bifidobacterium* might offer increased protection against colon cancer. Considering that the high-risk subjects were determined by their diets, results from this study indicated that the population of bifidobacteria is diet-related, where they favourably grow in the presence of red-meat. Such a correlation is not new, as a previous study also reported that the population of *B. infantis* was higher in subjects consuming a Western diet than those on a native Japanese diet [40]. This could be a matter of concern, because bifidobacteria are often taken as a probiotic supplement with anticarcinogenic effects, and should not exhibit colon cancer potentials. More studies are needed for in-depth understanding on this matter, to ensure that the detrimental effects of bifidobacteria do not outweigh their widely claimed-benefits.

Another study on inulin also reported such controversial findings. It has been highly publicized that diets high in red meat and low in fiber would increase the risks of colon cancer. Mutanen *et al.* [41] conducted a study to determine whether such a claim would be scientifically sound, by feeding rats a high-beef diet with or without oat, rye-wheat bran and inulin. Although the rye-bran group showed lowest number of polyps in the distal small intestine, the authors found that the inulin group was close to the beef group and was significantly different (P<0.05) from the rye-bran group. In addition, the number of animals bearing tumours in the colon and caecum was 89% in the beef group and 100% in the inulin group. In order to determine a possible explanation for this, the authors measured mucosal levels of a target molecule for APC protein, the β-catenin. A higher concentration of β-catenin has been associated with malfunction of the APC protein, where β-catenin could stimulate gene expression in the nuclease. Mice fed the inulin diet was found to have the highest level of cytosolic ß-catenin in additional to the lowest number of adenomas. The authors postulated that the inulin diet promoted the formation of intestinal tumors via increasing the level of cytosolic ß-catenin. Other contradictory results on the use of prebiotics on colon cancer have also been reported over the years and are presented in Table 1.

## 5. Conclusions

The use of probiotics and prebiotics to prevent colon cancer has gained much attention due to positive outcomes from in-vivo and molecular studies. Various mechanisms have been proposed including its anticarcinogenic effects, antimutagenic properties, modification of differentiation processes in tumor cells, production of short chain fatty acids, and alteration of tumor gene-expressions. Despite all the positive findings, other researches have also reported insignificant colon cancer protective effects. However, the increased interest in these areas demonstrated the need for further evaluation to better understand the exact mechanisms involved, and to generate uncontroversial experimental evidence.

## Figures and Tables

**Table 1.** Insignificant protective role of prebiotics on colon cancer

Product type Dose (vol/day) Study period	No. of subjects Subject types Age (years)	Study design	Results and conclusions	References
Wheat bran	≥1 confirmed colorectal adenomas removed within 3 mths	Randomized	Dietary supplement of wheat-bran fiber did not protect against recurrent colorectal adenomas. At least 1 adenoma detected in 47.0% of high-fiber group and 51.2% of low-fiber group	[42]
High amount: 13.5 g/day or Low amount: 2.0 g/day	≥1 confirmed colorectal adenomas removed within 3 mths			
36 months	40–80 years old			
Oat-bran and wheat-bran	45 (31 male and 14 female)	Randomized	Colonic biopsies taken before and after the intervention showed no difference in the index of thymidine colonic-crypt-cell labeling, thymidine-labeling pattern, or nuclear aberrations between oat and wheat brans. Difference between polyp and non-polyp subjects was insignificant.	[43]
16.4 g fiber/day	History of colonic adenomas			
2 weeks	49 nonpolyp subjects (26 male and 23 female) Normal coloscopy within 24 mths 51–61 years old			
Low fat (20% of total calories), high-fiber (18 g dietary fiber/1000kCal/day) and 3.5 servings of fruits and vegetables per 1000 kcal	1905 subjects (male and female) ≥ confirmed colorectal adenomas removed within 6 mths	Randomized	39.7% from the treatment group and 39.5% from the control group experienced at least one recurrent adenoma. Adopting a diet that is low in fat and high in fiber, fruits, and vegetables did not influence the risk of recurrence of colorectal adenomas.	[44]
4 years	≥35 years old			
Ispaghula husk (given as 3.5 of orange flavored effervescent granules dissolved in water)	665 patients with ≥2 adenomas or 1 adenoma of > 5mm in diameter	Randomized, double-blind, placebo controlled	Patients in the fiber group had a 45% increase in recurrent adenomas relative to the placebo group after 3 years {p = 0.04}. Supplementation with ispaghula husk increased the risk for recurrent adenoma.	[45]
3 years	35–75 years old			
